# Determining the Maximum Cumulative Ratios for Mixtures Observed in Ground Water Wells Used as Drinking Water Supplies in the United States

**DOI:** 10.3390/ijerph8124729

**Published:** 2011-12-19

**Authors:** Xianglu Han, Paul S. Price

**Affiliations:** Toxicology and Environmental Research and Consulting, The Dow Chemical Company, 1803 Building, Midland, MI 48674, USA; Email: xhan2@dow.com

**Keywords:** cumulative, risk assessment, exposure, mixtures, groundwater, Hazard Index, MCR

## Abstract

The maximum cumulative ratio (MCR) developed in previous work is a tool to evaluate the need to perform cumulative risk assessments. MCR is the ratio of the cumulative exposures to multiple chemicals to the maximum exposure from one of the chemicals when exposures are described using a common metric. This tool is used to evaluate mixtures of chemicals measured in samples of untreated ground water as source for drinking water systems in the United States. The mixtures of chemicals in this dataset differ from those examined in our previous work both in terms of the predicted toxicity and compounds measured. Despite these differences, MCR values in this study follow patterns similar to those seen earlier. MCR values for the mixtures have a mean (range) of 2.2 (1.03–5.4) that is much smaller than the mean (range) of 16 (5–34) in the mixtures in previous study. The MCR values of the mixtures decline as Hazard Index (HI) values increase. MCR values for mixtures with larger HI values are not affected by possible contributions from chemicals that may occur at levels below the detection limits. This work provides a second example of use of the MCR tool in the evaluation of mixtures that occur in the environment.

## 1. Introduction

The maximum cumulative ratio (MCR) is a useful tool in the evaluation of the need to perform cumulative risk assessments (CRAs) for non-carcinogenic effects [1]. MCR is defined as the ratio of the cumulative exposure (C) to multiple chemicals to the largest exposure from a single chemical (M). Calculation of the MCR requires a method to normalize exposures across chemicals. Example of such approaches are the hazard quotient/hazard index (HQ/HI) and various systems of toxicity equivalents (TEQs). Determining when CRAs are needed is important to risk managers since performing CRAs can be resource-intensive and time-consuming. Larger MCR values indicate a greater need for CRAs and smaller values indicate less need. 

The HI/HQ approach can be used to calculate MCR values when permitted doses (PDs) have been established for the chemicals and estimates of the doses from an individual’s exposure can be determined. The HQ is defined as an individual’s dose of a chemical divided by the PD:


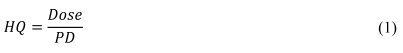


The dose of a chemical can be based on screening exposure assumptions and reported concentrations of multiple chemicals in a sample of a media (air, water, surface or food) or estimates of doses received by an individual in a cumulative exposure assessment from multiple sources. The PD is the dose below which an individual is believed to be protected against the chronic non-carcinogenic effects of the chemical. Examples of such doses are reference doses (RfDs), population adjusted doses (PADs), acceptable daily intakes (ADIs), derived no-effect levels (DNELs), and minimal risk level (MRLs). HQs of the components are summed to provide a measure of cumulative exposure, the Hazard Index (HI): 


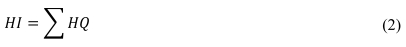


The HQ can also be viewed as a toxicity-normalized measure of exposure to a common “index chemical”. The HI can be used as a measure of C and the maximum HQ of a mixture’s components can be used as a measure of M: 


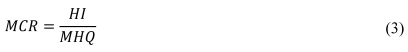


where MHQ is the maximum of multiple HQ values calculated for an individual’s exposures to multiple chemicals.

Because of the way MCR is defined, when dose additivity is assumed the MCR value for an individual is bounded by 1 and the number of chemicals considered in the assessment (n). An MCR value of 5 indicates that 80% of the individual’s HI would be missed if a chemical-by-chemical method is used to assess the individual instead of a CRA. An MCR value of 1.25 indicates the missing portion is 20%. An MCR value of less than 2 is an indication that one compound provides the majority (>50%) of the HI for an individual’s estimated exposure. 

The findings from the initial application of MCR to mixtures of plant protection products (PPPs) measured in samples of surface water were that:

- In mixtures with five to 29 detected PPPs (with a mean of nine detections), MCR values (range of 1.0–4.0 with a mean of 1.8) were much smaller than the number of detected compounds and were inversely related to the toxicity of the mixtures.
- Mixtures with HI values greater than 1 had mean MCR values of 1.3 [1].

The purpose of this work is to determine if the patterns of MCR values observed in surface water samples [1] also occur in mixtures of chemicals measured in other datasets of environmental samples. The specific goals are to determine: (1) the values of *n*, HI and MCR for an additional dataset, (2) explore the relationship between MCR, *n*, and HI in the new dataset, and (3) to investigate the impact of non-detects on the MCR values. 

In this paper, we report the results of the application of the MCR to the mixtures of chemicals reported to occur in water sample of the 1993–2007 survey of ground water performed by the United States Geological Survey (USGS) [2,3,4,5]. These mixtures were observed in well water samples taken from public water systems across the U.S. The samples were analyzed for a wide variety of chemicals including PPPs, volatile organic compounds (VOCs), metals, and other inorganics [3]. 

There were multiple reasons for investigating the USGS ground water dataset. Firstly, the dataset includes a large number of analytes and detections, there are PDs available for most of the compounds, and there are a relatively large number of samples. Secondly, the source, toxicity and composition of the reported mixtures are different from the mixtures investigated in our initial publication [6]. The sources of the water samples are ground water wells from productive aquifers. These wells are deep and draw water from large areas. In addition, the wells are often required to be sited away from known point-sources of potential contamination [7]. The mixtures of chemicals were of greater toxicological concern (HI values were larger) than the mixtures of PPPs reported to occur in surface water samples examined in the earlier study. Approximately one in five of the mixtures measured in the ground water samples contained one or more compounds at concentrations that raise health concerns [2]. In the PPP dataset this was true for only 0.5% of the mixtures [1]. In addition, the ground water samples were analyzed for a much wider range of compounds than the surface waters. It should be noted that because neither survey analyzed for all compound present in the samples and because of different analytes were measured in the two surveys, no conclusion can be drawn on the relative toxicities of the surveyed bodies of water. This paper focuses on the mixtures of the compounds measured in the two surveys. Finally, the source of the compounds with the largest hazard quotients (HQs) is different. In the ground water data, the compounds of greatest toxicity are inorganic compounds that could be the result of anthropogenic activity or could naturally occur. In the earlier study the source of the PPPs were the agricultural practices at the time of the sampling (1990s). 

## 2. Experimental Section

### 2.1. Derivation of MCR

The calculation of MCR values was based on the approach described in Price and Han (2011) [1] and the introduction of this paper. In this paper, MCR is calculated based on the assumption that dose additivity applies to all chemicals in the mixtures. This screening assumption would be revisited in later tiers of a CRA [8,9].

### 2.2. Data Treatment and Reduction

The groundwater dataset consists of mixtures observed in 932 samples. The measured compounds include major ions (11), trace elements (23), PPPs and PPP metabolites/degradates (83), and volatile organic compounds (85). The analytical methods varied across the samples and no one sample was analyzed for all 200 compounds. Many of these compounds were rarely detected and 58 were never detected in any sample. In this analysis, we have assumed that chemicals not detected in any of the samples do not occur in the sampled wells. As a result, this paper focuses on the contributions of remaining 142 compounds. 

As discussed above, all samples had missing values for some analytes. In performing the assessment, we excluded samples where compounds, known to be important contributors to HI, were not measured. The importance of measuring specific compounds was determined by ranking all the chemicals based on the means of their corresponding HQs in the mixtures and then only evaluating mixtures that included the chemicals that make the largest contributions. Two possible options were investigated: (1) excluding data from samples if any of the top three chemicals (with largest mean HQs) were not measured and (2) excluding data from samples if any of the top six chemicals were not measured. As shown below, application of either criterion resulted in similar distributions of MCR values in the dataset. Option 1 was used since it excludes less data. In addition, in order to avoid producing estimates of MCR that are biased by a mixture that has only too few components, all mixtures containing less than 5 compounds that occur at detectable levels were removed from the dataset. 

There are a large number of samples where a number of analytes have levels below the detection limits (non-detects or NDs). This presents a challenge for characterizing cumulative exposures using monitoring data. While risk assessors should not assume that non-detected compounds are absent from samples [1], inclusion of NDs could introduce large uncertainties, especially when there are a large number of NDs that could drive the estimates of the toxicity of the mixture and the MCR values. In order to investigate the impact of non-detects on HI and MCR values, the data were analyzed using two assumptions, Case 1 where concentrations of NDs were set as 0, and Case 2 where the concentrations are assumed to be equal to the detection limit (DL) divided by 2^0.5^ [10]. This method for treating NDs is one of the most commonly used methods and is better than assuming ND are equal to DL/2 for lognormally distributed data [10]. While not shown, the concentrations of many of the compounds are generally lognormally distributed. HI and MCR values generated in Cases 1 and 2 were compared to determine the impact of NDs. 

### 2.3. Permitted Doses

PDs for chronic oral non-cancer health effects are available from the U.S. Environmental Protection Agency (EPA), Agency for Toxic Substances and Disease Registry (ATSDR), and other sources. The highest priority used for selecting the values of the PDs used in this study was given the chronic RfDs set by the EPA for non-PPPs. For PPPs the chronic Population Adjusted Doses (PADs) were used. When a chronic standard was not available for certain PPPs we have used the acute PAD. 

If these standards were not available, PDs from other national regulatory agencies (such as ATSDR) were used. Lower priority was given to PDs set by a State and to provisional values set by these agencies. One chemical, chloromethane, did not have an established oral RfD but an RfC of 0.09 mg/m^3^ was available. An equivalent oral dose of the RfC was estimated based on the assumptions of a breathing rate of 20 m^3^/day, a lung clearance of 40%, and a body weight of 60 kg. 

In this study, exposures to the mixtures in the samples are assumed to occur on a chronic basis and the doses are conservatively determined by assuming a drinking water consumption rate of 2 liters of water per day, 100% oral absorption, and a body weight of 60 kg. These assumptions are typically used to conduct safety assessments for chemicals in water; however, it is important to note that the water samples were taken prior to any treatment and thus do not reflect actual exposures to the mixtures.

The principle for choosing or developing standards in this work is different from that used in the USGS publication [4]. The approach used by the USGS was to compare the concentrations of the contaminants to the corresponding human health benchmarks including maximum contaminant level (MCL) developed by the USEPA for regulating drinking water and non-regulatory health-based screening level (HBSL) that were developed by the USGS and other organizations. The USGS approach was not used since many of the standards used are based on carcinogenic effects and often assume that only 20% of the intake of a contaminant occurs from water [5]. Because our analysis focuses on the effects from exposure to the mixture of chemicals from a specific source and not the cumulative risks from all sources of the contaminants, it is inappropriate to include the source-apportionment factor. 

### 2.4. Statistical Analyses

Two methods were used to investigate the relationship between MCR and HI. The first method is a scatter plot in which the MCR values of the mixtures were plotted against the corresponding HI values. In the second method, the MCR values of mixtures were ranked based on the HI values of the mixtures and three portions of mixtures were identified that have HI values falling in the 49–51^st^, 94–96^th^, and 98–100^th^ centiles of HI values. The MCR values for these three groups were determined. The goal was to characterize the range of MCR values that occur in mixtures that have typical (50^th^ centile, high-end values 95^th^ centile, and upper bound values of HI 99^th^ centile). We chose the 2% of the population around these percentiles to provide a reasonable group size (11 values). The minimum, maximum, and means of these three groups were reported. Statistical differences between the three groups were determined as described below. These analyses were performed separately for Case 1 and Case 2.

The relationship between the number of chemicals in a mixture (*n*) and the HI and MCR values was also investigated to determine if mixtures with larger values of n had different toxicities and MCR values. In Case 1, the concentrations of NDs are set as 0, therefore n is defined as the number of detects in the samples. In Case 2, n is defined as the number of analytes. Two tests for trends between values of n and HI and MCR values were performed: one using data on individual mixtures and the other looking at median HI and median MCR values of mixtures grouped by n. Medians were only calculated for values of n where there were at least 5 values. 

The nonparametric Wilcoxon tests were performed for the comparisons of the MCR values for the samples of the 49–51^st^, 94–96^th^, and 98–100^th^ centile ranges of HIs, the comparison between Cases 1 and 2, and the comparisons of MCR values from mixtures with HI less than and greater than 1. The relationships between MCR and HI and the relationship between n and the HI and MCR values were evaluated using the nonparametric correlation test—Kendall’s rank correlation (correlation coefficient τ) in the statistical software JMP^®^ (JMP^®^ Pro 9.0.1, SAS Institute Inc.). JMP^®^ was also used to perform all other statistical tests. Data reduction, MCR calculation and trend analysis were conducted in Microsoft Office (Excel^®^) 2007.

## 3. Results and Discussion

### 3.1. Data Description and Reduction

The impacts of the two exclusion criterion (requiring measurements of the top three or top six compounds) on MCR values are presented in Table 1. This table presents the MCR values for three subgroups of mixtures with typical, high end, and upper bound values of HI. These subgroups were created by ranking the mixtures based on their HI values and selecting the mixtures with HI values that fell in the 49–51^st^, 94–96^th^, and 98–100^th^ centiles. The mean MCR values are determined for each of the three groups. These analyses were performed assuming that concentrations of NDs were equal to DL/2^0.5^. 

The results in Table 1 indicate that the two approaches produce similar MCR values. The less stringent requirement on missing values in the top three compounds was used since this criterion allows the use of data from more samples. A total of 627 samples of the original 932 met this criterion. An additional 9 samples were excluded because they had less than 5 detected components giving a final dataset of 618 samples. The total number of analytes, detects, and NDs for the 627 samples are given in Table 2.

**Table 1:** Impact of the two exclusion criteria (missing value in top three or top six compounds) on mean MCR values in three portions of the mixtures. Portions are determined by ranking mixtures and selecting mixtures that fall within three ranges of HI centiles (49–51, 94–96, and 98–100 centiles).

Options	Mixtures center	Centiles of HI values		
		49–-51^st^	94–96^th^	98–100^th^
Top 3	627	2.5	1.5	1.2
Top 6	437	2.5	1.6	1.2

**Table 2:** The number of analytes and detects in the final set of 618 mixtures.

Statistics	Minimum	Maximum	Mean
Number of detects	5	34	16
Number of nondetects	28	104	82
Number of analytes	43	112	98

### 3.2. Permitted Doses

PDs were found for 114 of the 144 contaminants detected in one or more of the groundwater samples. Fluoride is the only ion, of the nine major ions, with an available PD. The remaining ions are calcium, magnesium, sodium, potassium, chloride, bromide, sulfate, and silica. PDs were identified for 22 of 23 trace elements, 81 of 83 PPPs, and 57 of 85 volatile organic compounds (Table 3). In the case of metabolites of PPPs we have assumed that the metabolites have the toxicities of the parent compounds. The sources of these PDs are listed in Table 4. When the maximum contaminant level (MCL) was available, the noncancer toxicity standard was used. Eight of the 30 chemicals with no available PDs are essential elements that are not expected to have adverse biological effects at the levels observed in the samples. The remaining 22 chemicals occur infrequently (<2% of the samples) and omitting their contributions is not anticipated to have a significant effect on the distribution of HI or MCR values. Table 3 also includes PD for 47 compounds that were measured but never detected. These values were used in the development of the exclusion criteria described in Section 3.1.

**Table 3:** Permitted doses (PDs) used in this study.

Chemical	Source Code ^1^	PD (mg/kg/day)	Basis	Chemical	Source Code	PD (mg/kg/day)	Basis
1,1,1-Trichloroethane	1	2	RfD	Dichloromethane	1	0.06	RfD
1,1,1, 2-Tetrachloroethane	1	0.03	RfD	Dieldrin	2	0.00005	RfD
1,1,2-Trichloro-1,2,2-trifluoroethane	1	30	RfD	Diethyl ether	1	0.2	RfD
1,1,2-Trichloroethane	1	0.004	RfD	Diisopropyl ether	10	0.1	RfD
1,1-Dichloroethane	8	0.07	RfD	Dinoseb	1	0.001	RfD
1,1-Dichloroethene	1	0.05	RfD	Diuron	2	0.003	RfD
1,2,3-Trichloropropane	1	0.004	RfD	EPTC	2	0.0025	RfD
1,2,4-Trichloro-Benzene	1	0.01	RfD	Ethoprop	2	0.0001	RfD
1,2,4-Trimethylbenzene	10	0.05	RfD	Ethyl methyl ketone	1	0.6	RfD
1,2-Dibromo-3-chloropropane	10	0.0002	RfD	Ethylbenzene	1	0.1	RfD
1,2-Dibromoethane	1	0.009	RfD	Fluometuron	2	0.005	RfD
1,2-Dichlorobenzene	1	0.09	RfD	Fluoride	1	60	RfD
1,2-Dichloropropane	14	0.09	MRL	Hexachloro-butadiene	9	6.70E-05	RfD
1,4-Dichlorobenzene	14	0.07	MRL	Isopropylbenzene	1	0.1	RfD
2,4-D	2	0.005	RfD	Lead	7	0.0005	MCL
2,6-Diethylaniline^2^	2	0.006	RfD	Lindane	1	0.0003	RfD
2-Chloro-4-isopropylamino-6-amino-s-triazine	2	0.0018	RfD	Linuron	2	0.0077	RfD
Acetochlor	1	0.02	RfD	Lithium	10	0.02	RfD
Acetone	1	0.9	RfD	Manganese	1	0.14	RfD
Acrylonitrile	1	0.002	RfD	Methyl parathion	2	0.00002	RfD
Alachlor	2	0.01	RfD	Methyl tert-butyl ether	10	0.01	RfD
Aldicarb	3	0.00027	RfD	Methyl tert-pentyl ether	10	0.04	RfD
Aldicarb sulfone	3	0.00027	RfD	Metolachlor	2	0.1	RfD
Aldicarb sulfoxide	15	0.00027	RfD	Metribuzin	2	0.013	RfD
alpha-HCH	16	0.008	RfD	Molinate	17	0.001	RfD
Aluminum	10	1	RfD	Molybdenum	1	0.005	RfD
Antimony	1	0.0004	RfD	*m*- + *p*-Xylene	1	0.2	RfD
Arsenic	1	0.0003	RfD	Naphthalene	1	0.02	RfD
Atrazine	2	0.0019	RfD	Nickel	1	0.02	RfD
Barium	1	0.2	RfD	Nitrate	1	1.6	RfD
Bentazon	2	0.03	RfD	Nitrite	1	0.1	RfD
Benzene	1	0.004	RfD	Norflurazon	2	0.015	RfD
Beryllium	1	0.002	RfD	o-Xylene	1	0.2	RfD
Boron	1	0.2	RfD	p,p'-DDE	18	0.0005	RfD
Bromacil	2	0.1	RfD	Picloram	2	0.2	RfD
Bromobenzene	1	0.008	RfD	Prometon	2	0.05	RfD
Bromochloro Methane	10	0.04	RfD	Propoxur	2	0.005	RfD
Bromodichloro Methane	1	0.02	RfD	Selenium	1	0.005	RfD
Bromoxynil	2	0.015	RfD	Silver	1	0.005	RfD
Butylate	2	0.05	RfD	Simazine	2	0.0018	RfD
Cadmium	1	0.0005	RfD	Strontium	1	0.6	RfD
Carbaryl	2	0.1	RfD	Styrene	1	0.2	RfD
Carbofuran	2	0.00006	RfD	Tebuthiuron	2	0.07	RfD
Carbon disulfide	1	0.1	RfD	Terbacil	2	0.013	RfD
Chloramben methyl ester	4	0.014	RfD	Tetrachloro ethene	1	0.01	RfD
Chlorobenzene	1	0.02	RfD	Tetrachloro methane	1	0.004	RfD
Chloromethane	1	0.01	RfD	Thallium	10	0.00008	RfD
Chlorpyrifos	2	0.00003	RfD	Toluene	1	0.08	RfD
Chromium	1	0.003	RfD	trans-1,2-Dichloroethene	1	0.02	RfD
*cis*-1,2-Dichloroethene	1	0.002	RfD	Tribromomethane	1	0.02	RfD
Clopyralid	19	0.15	RfD	Trichloroethene	13	0.05	TDI
Cobalt	10	0.06	RfD	Trichlorofluoro-methane	1	0.3	RfD
Copper	12	0.01	RfD	Trichloromethane	1	0.01	RfD
Cyanazine	5	0.00026	RfD	Uranium (natural)	7	30	MCL
DCPA	2	0.01	RfD	Vanadium	11	0.01	MRL
Diazinon	2	0.0002	RfD	Vinyl chloride	1	0.003	RfD
Dibromochloro- methane	1	0.02	RfD	Zinc	1	0.3	RfD
Dichlorodifluoromethane	1	0.2					

^1^ Source code is given in Table 4.

**Table 4:** Sources of permitted doses used in this study.

Source code	Source
1	USEPA Integrated Risk Information System.
	http://cfpub.epa.gov/ncea/iris/index.cfm?fuseaction=iris.showSubstanceList.
2	USEPA Office of Pesticide Programs Pesticide Reregistration Status.
	http://www.epa.gov/opp00001/reregistration/status.htm
3	http://www.regulations.gov/#!documentDetail;D=EPA-HQ-OPP-2005-0163-0249
4	http://www.consumersunion.org/pdf/fqpa/ReportCard_appendix1.pdf
5	Minnesota Department of Health. Health Risk Limits for Groundwater 2008 Rule Revision Health Risk Assessment Unit, Environmental Health Division.
	http://www.health.state.mn.us/divs/eh/risk/guidance/gw/cyanazine.pdf
6	Agency for Toxic Substances and Disease Registry. Toxicological Profiles
	http://www.atsdr.cdc.gov/ToxProfiles/tp1.pdf
7	USEPA Drinking Water Standards and Health Advisories Table.
	http://water.epa.gov/action/advisories/drinking/upload/dwstandards2011.pdf
8	http://www.mass.gov/dep/water/drinking/standards/11dichle.htm
9	New York ADI. www.epa.gov/Region5/glic/pdfs/ny_hh_182_w_03121998.pdf
10	www.tceq.state.tx.us/assets/public/remediation/rrr/rrrupdate2008.xls
11	ATSDR, 2009. http://www.atsdr.cdc.gov/toxprofiles/tp58.pdf
12	http://www.atsdr.cdc.gov/ToxProfiles/tp132-c8.pdf
13	Provisional TDI of 0.05 mg/kg/day from the National Institute for Public Health and the Environment (RIVM -- Dutch). http://toxnet.nlm.nih.gov/cgi-bin/sis/search/f?./temp/~l72TEe:1
14	http://toxnet.nlm.nih.gov/cgi-bin/sis/search
15	http://www.regulations.gov/#!documentDetail;D=EPA-HQ-OPP-2005-0163-0250
16	http://www.regulations.gov/#!documentDetail;D=EPA-HQ-OPP-2006-0034-0002
17	http://www.regulations.gov/#!documentDetail;D=EPA-HQ-OPP-2003-0397-0003
18	http://www.regulations.gov/#!documentDetail;D=EPA-HQ-OW-2007-0068-0182
19	http://www.regulations.gov/#!documentDetail;D=EPA-HQ-OPP-2009-0092-0006

### 3.3. Drivers of Mixture Toxicity

As discussed above, a significant number of mixtures measured in the samples were reported to have health concern [3]. In this analysis the percentage of the mixtures with HI values greater than 1 ranged between 26% and 34% depending on how NDs were assessed. Table 5 presents the six chemicals with the largest mean HQs in the 618 mixtures. On average, these top six chemicals contribute 74% of the mixtures’ HI values.

**Table 5:** Chemicals with the highest average hazard quotient (HQ) in the 618 mixtures and their cumulative contributions to the mean HI of the mixtures.

Chemical	Mean HQ in the 618 mixtures (Case 2)	Cumulative percentage of mixtures’ mean HI (Case 2)
Arsenic	0.362	33%
Fluoride	0.217	52%
Uranium	0.098	61%
Lead	0.059	66%
Lithium	0.040	70%
Strontium	0.040	74%

### 3.4. MCR Results

**Figure 1:**
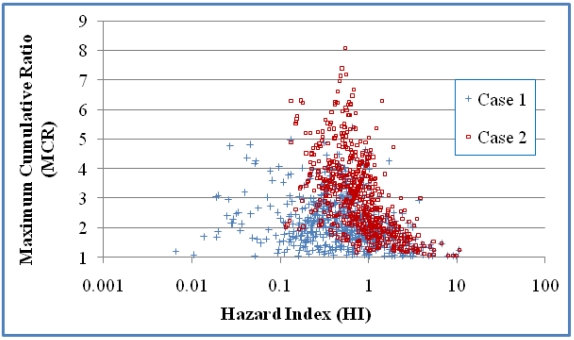
A scatter plot of the HI and MCR values for the mixtures in the 618 mixtures. Case 1 assumes that NDs have a concentration of 0 and Case 2 assumes that NDs have concentrations of DL/2^0.5^. Kendall correlation coefficients indicate a statistically significant negative correlation between MCR and HI for both cases (τ = −0.2132 and *p* < 0.0001 in Case 1; τ = −0.4362 and *p* < 0.0001 in Case 2).

**Figure 2:**
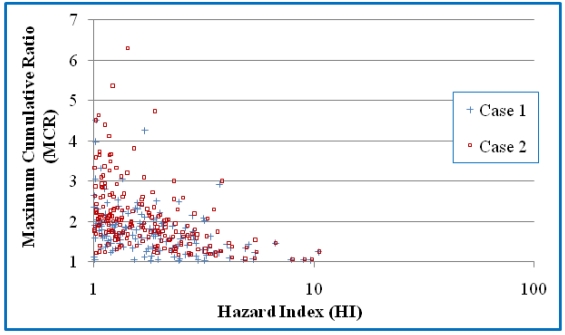
A scatter plot of the HI and MCR values for the mixtures with HI values greater than 1. Case 1 assumes that NDs have a concentration of 0 and Case 2 assumes that NDs have concentrations of DL/2^0.5^.

MCR and HI values are determined for each of the mixtures. Figure 1 and Figure 2 present scatter plots of the mixtures and those mixtures with HI values greater than 1 respectively. Values are presented for both Cases 1 and 2. Kendall correlation coefficients showed negative correlation between HI and MCR for Cases 1 and 2 (*p* < 0.0001 in both cases). Comparison of Cases 1 and 2 indicates that the different treatments on NDs have a large influence on MCR values of mixtures with smaller HIs (Figure 1) but have little impact on MCR values of mixtures with HI greater than 1 (Figure 2). 

Table 6, Table 7 and Table 8 present the HI and MCR values for all mixtures, mixtures with HI greater and less than 1, and three subgroups of the mixtures respectively. Separate results are presented for Cases 1 and 2. Because of the contributions of the HQs associated with the NDs in Case 2, the HI values in Case 2 are always higher than those in Case 1 (0.19 higher on average for all mixtures). The MCR values in Case 2 are typically but not always higher than Case 1 (Table 6). 

**Table 6:** HI and MCR values in the final dataset of 618 mixtures. Statistical significance was shown for the differences in HI and MCR values between Cases 1 and 2 (*p* < 0.0001, Wilcoxon test). Case 1 assumes that NDs have concentrations of 0 and Case 2 assumes that NDs have concentrations of DL/2^0.5^.

Cases	HI Values			MCR Values		
	Minimum	Maximum	Mean	Minimum	Maximum	Mean
Case 1	0.001	10.4	0.86	1.03	5.4	2.2
Case 2	0.116	10.6	1.05	1.05	8.1	3.1

Dividing the 618 mixtures into two subgroups (those with HI less than 1 or HI greater than 1), shows that 158 (26%) and 208 (34%) of the mixtures have HI values greater than 1 in Cases 1 and 2, respectively. For both subgroups, the MCR values in Case 2 are higher than those in Case 1 but the mean difference is 1.3 for the mixtures with HI values less than 1 and 0.4 for mixtures with HI values greater than 1 (Table 7). 

**Table 7:** Comparison of MCR values for mixtures with HI values greater or less than 1. For both groups of mixtures the MCR values in Case 2 are significantly higher than those in Case 1 (*p* < 0.0001 in Wilcoxon test). Case 1 assumes NDs have concentrations of 0 and Case 2 assumes that NDs have concentrations of DL/2^0.5^. Min: Minimum; Max: Maximum.

Cases	Mixtures with HI <1				Mixtures with HI >1			
	% of all mixtures	Min MCR	Max MCR	Mean MCR	% of all mixtures	Min MCR	Max MCR	Mean MCR
1	74%	1.03	5.4	2.3	26%	1.0	4.5	1.7
2	66%	1.16	8.1	3.6	34%	1.1	6.3	2.1

MCR values in Cases 1 and 2 were also compared by choosing three portions of mixtures (49–51^st^, 94–96^th^, and 98–100^th^ centile ranges of the HI values of the mixtures). There are only small differences between Cases 1 and 2 in these groups and these differences had no statistical significance based on Wilcoxon test (Table 8). 

**Table 8:** Comparison of the MCR values of three portions of mixtures in Cases 1 and 2. No statistical differences were found between the two cases for the three portions of samples in Wilcoxon test. The three portions of mixtures were chosen on the basis of HI (mixtures with HI values falling into 49–51^st^, 94–96^th^, and 98–100^th^ centile ranges respectively). Case 1 assumes that NDs have concentrations of 0 and Case 2 assumes that NDs have concentrations of DL/2^0.5^. Min: minimum; Max: maximum.

Case	49–51^st^ Centile			94–96^th^ Centile			98–100^th^ Centile		
	Min MCR	Max MCR	Mean MCR	Min MCR	Max MCR	Mean MCR	Min MCR	Max MCR	Mean MCR
1	1.86	4.0	2.8	1.2	2.1	1.6	1.04	1.5	1.2
2	1.52	3.3	2.6	1.2	2.2	1.5	1.05	1.5	1.2

**Figure 3:**
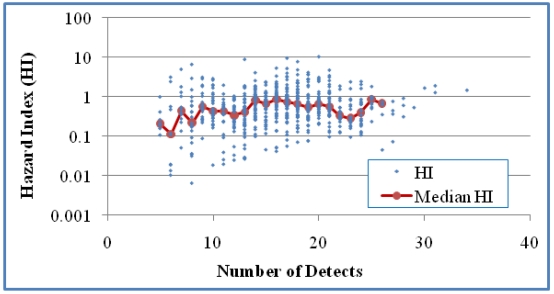
The relationship between HI and the number of detects in the samples for Case 1 (618 mixtures). A positive correlation was shown based on all mixtures (Kendall’s τ = 0.085 and *p* < 0.01) but not median HI of grouped mixtures for groups with at least five values (τ = 0.0913 and *p* > 0.05).

**Figure 4:**
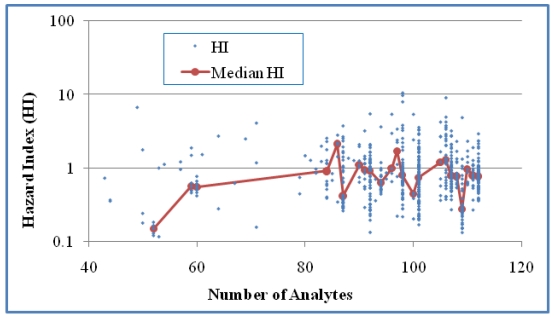
The relationship between HI and the number of analytes in the samples for Case 2 (618 mixtures). No statistical significance was shown for this correlation either based on all samples (Kendall’s τ = 0.0196 and *p* > 0.05) or median HI of grouped samples for groups with at least five values (τ = 0.0554 and *p* > 0.05).

The relationship between HI and the number of detects in the mixtures was studied for Case 1 (Figure 3). For Case 2 (Figure 4), the number of analytes was used for examining this relationship since NDs were involved in the calculation of MCR values. For Case 1 there was a three-fold increase in HI when n was increased from 13 to 25, but no clear trend above an n of 25. The trend of increased HI values as n increased was statistically significant when based on HI values of individual mixtures (*p* < 0.0001), but not when based on the medians of the HI values of grouped mixtures (*p* > 0.05). No statistical significance was shown for this correlation in Case 2 either based on HI values of individual mixtures or median HI values of grouped mixtures for groups with at least five values (*p* > 0.05). When grouping mixtures based on the same number of detects (Figure 3) or analytes (Figure 4), we obtained medians from groups of at least five values in order to generate more reliable medians for trend analysis. These observations suggest that an increase in the number of detected analytes is weakly associated with an increase in HI but an increase in the number of analytes is not associated with larger HI values. 

**Figure 5:**
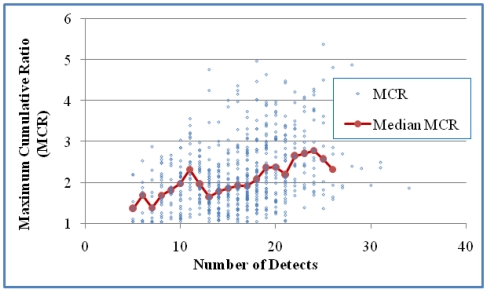
The relationship between MCR and the number of detects in the samples for Case 1 (618 mixtures). A positive correlation was shown either based on all mixtures (Kendall’s τ = 0.2511 and *p* < 0.0001) or median MCR of grouped mixtures for groups with at least five values (τ = 0.6826 and *p* < 0.0001).

**Figure 6:**
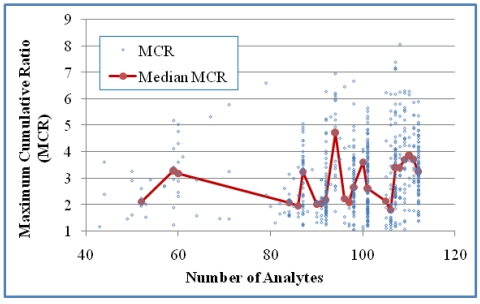
The relationship between MCR and the number of analytes in the samples for Case 2 (618 mixtures). A positive correlation was shown based either on all mixtures (Kendall’s τ = 0.1501 and *p* < 0.0001) or on median MCR values of grouped mixtures for groups with at least five values (τ = 0.3202 and *p* < 0.05).

The relationship between MCR and n for the two cases are presented in Figure 5 and Figure 6. A positive correlation was shown for Case 1 either based on all mixtures (*p* < 0.0001) or median MCR of grouped mixtures for groups with at least five values (*p* < 0.001). A positive correlation for Case 2 was shown based on all mixtures (*p* < 0.0001) and for median values of grouped mixtures (*p* < 0.05). This suggests that increases in both the number of detects and the number of analytes are indicators of modest increases in MCR values. The MCR values for mixtures measured in samples with 5–10 detects ranged from 1.0 to 2.0 while the MCR values for mixtures measured in samples with 15–25 detects had a wider range (1.0 to 5.0). 

### 3.5. Discussion

The issue of cumulative exposures to chemicals has been drawing increasing attention in the fields of toxicology and risk assessment. The MCR is a tool that can help evaluate the need forCRAs. Smaller MCR values indicate that a single chemical is driving the total toxicity resulting from cumulative exposures. 

Price and Han [1] previously investigated the range of HI and MCR values for a group of PPPs measured in surface water samples. In this paper, we have performed a similar analysis on a second dataset of organic and inorganic compounds in ground water wells used as drinking water supplies across the U.S. [3]. These samples differ in terms of source (ground water *versus* surface water), HI values (mean HI value of 0.87 *versus* 0.14 when setting NDs at 0), and the number and variety of analytes (a range of organic and inorganic compounds *versus* PPPs). The PPPs in the surface water samples resulted mainly from agricultural use of PPPs while the compounds in this study came from natural sources, uncontrolled waste disposal, as well as PPP use. The top six contributors to the HI values of mixtures are inorganic chemicals (Table 5) which may occur naturally or could result from human activity. 

Despite these differences, many of the findings in the first study are also observed in this dataset. The vast majority of mixtures in the ground water samples have MCR values below 5 (Figure 1) with an average MCR value of 2.2–3.1 (Table 6). These findings suggest that the HI values of most mixtures are dominated by just a few chemicals. Figure 1 and Figure 2 indicate that, as an overall trend, MCR values decrease as HI values increase. This trend is more obvious when only focusing on the mixtures with HI values greater than one. The trends suggest that mixtures with higher toxicity are in general dominated by the toxicity of the primary chemical and have less need for a CRA. 

**Figure 7:**
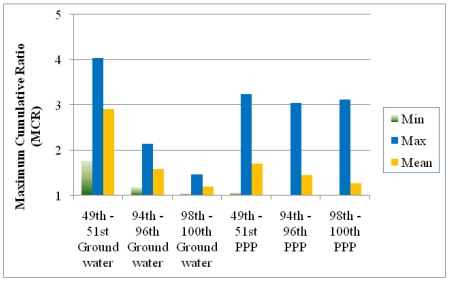
Comparison of the MCR values in three groups of the mixtures in the surface water samples analyzed for PPPs [1] and the results from this study (ground water samples). The three groups of mixtures were chosen on the basis of HI (mixtures with HI values falling into 49–51^st^, 94–96^th^, and 98–100^th^ centile ranges respectively). Min: minimum; Max: maximum.

Figure 7 presents the minimums, maximums, and means of MCR in mixtures with HI values falling in the 49–51^st^, 94–96^th^ and 98–100^th^ centile ranges of HI values. The data were taken from our original publication [1] and the new work presented here. The MCR values are higher for the mixtures with typical HI values. This may be a reflection of the fact that more compounds and more detects occurred in groundwater study. MCR values in the ground water study clearly decrease with increasing toxicity for both datasets. The average MCR values, as presented in yellow, are higher in mixtures with HI values near the median and 95^th^ centiles but smaller for mixtures with HI values in the top two centiles. In this dataset the maximum MCR values in the three groups also declined suggesting a stronger trend than that observed in the surface water data. 

## 4. Conclusions

This work provides further evidence for the finding that the toxicities of environmental mixtures are dominated by a relatively small number of components and mixtures of higher toxicity are frequently dominated by one component. This demonstrates the usefulness of MCR as a screening tool to help in determination of the need for CRAs. 
